# Computational Generation of Long-range Axonal Morphologies

**DOI:** 10.1007/s12021-024-09696-0

**Published:** 2025-01-10

**Authors:** Adrien Berchet, Remy Petkantchin, Henry Markram, Lida Kanari

**Affiliations:** Blue Brain Project, EPFL, Chemin des mines 9, 1202 Geneva, Switzerland

**Keywords:** Neuronal morphology, Axon synthesis, Steiner tree, Algebraic topology, Brain connectivity

## Abstract

Long-range axons are fundamental to brain connectivity and functional organization, enabling communication between different brain regions. Recent advances in experimental techniques have yielded a substantial number of whole-brain axonal reconstructions. While previous computational generative models of neurons have predominantly focused on dendrites, generating realistic axonal morphologies is more challenging due to their distinct targeting. In this study, we present a novel algorithm for axon synthesis that combines algebraic topology with the Steiner tree algorithm, an extension of the minimum spanning tree, to generate both the local and long-range compartments of axons. We demonstrate that our computationally generated axons closely replicate experimental data in terms of their morphological properties. This approach enables the generation of biologically accurate long-range axons that span large distances and connect multiple brain regions, advancing the digital reconstruction of the brain. Ultimately, our approach opens up new possibilities for large-scale in-silico simulations, advancing research into brain function and disorders.

## Introduction

The brain is a highly complex organ responsible for controlling essential functions such as sensory processing, cognition, memory, respiration, motor control, and language production (Ito et al., 2020; Adamovich et al., 2024; Iranmanesh et al., 2021). These functions rely on intricate computations within distinct brain regions and the transmission of signals between them. Long-range axons, also referred to as projecting axons, control this interregional communication (Liu et al., 2024; Shin et al., 2020), and numerous studies have highlighted their critical role in global neuronal circuitry (Zingg et al., 2014; Hilgetag & Zikopoulos, 2022; Mateus et al., 2024). Therefore, understanding the organization and connectivity of long-range axons is vital for deciphering brain function.

Recent advances in experimental imaging techniques, such as the sparse labeling technique, described in Economo et al. (2019) and Winnubst et al. (2019), overcome limitations of previous reconstruction efforts, such as electron microscopy (Helmstaedter et al., 2013; Kasthuri et al., 2015), and staining of local axons (Thomson & Armstrong, 2011; Blackman et al., 2014; Karube et al., 2017), enabling the reconstruction of complete long-range axonal morphologies that span multiple brain regions. However, the process remains time-consuming and costly, making it impractical to capture every detail of the biological neuronal circuits (Economo et al., 2019; Wang et al., 2018). In addition, experimental approaches face limitations in manipulating neuronal morphological properties to assess their effects on brain function. To overcome these challenges, numerical simulations of large-scale neuronal circuits, which we term as morphologically detailed circuits, are essential to gain deeper insights into brain organization and the distribution of tasks across different brain regions. These simulations should model a large number of neuronal morphologies, including both dendrites and axons, to establish complete circuit connectivity. A major challenge is generating realistic neuronal circuits in which the modeled morphologies exhibit local morphometric properties (e.g., branching patterns and section characteristics) that closely resemble those of experimentally reconstructed neurons. Although this work does not feature circuit simulations, it provides a new tool to synthesize multiple long-range axons accurately. This is fundamental for large-scale circuit simulations as these long-range axons enable the connection between brain regions.

**Fig. 1:**
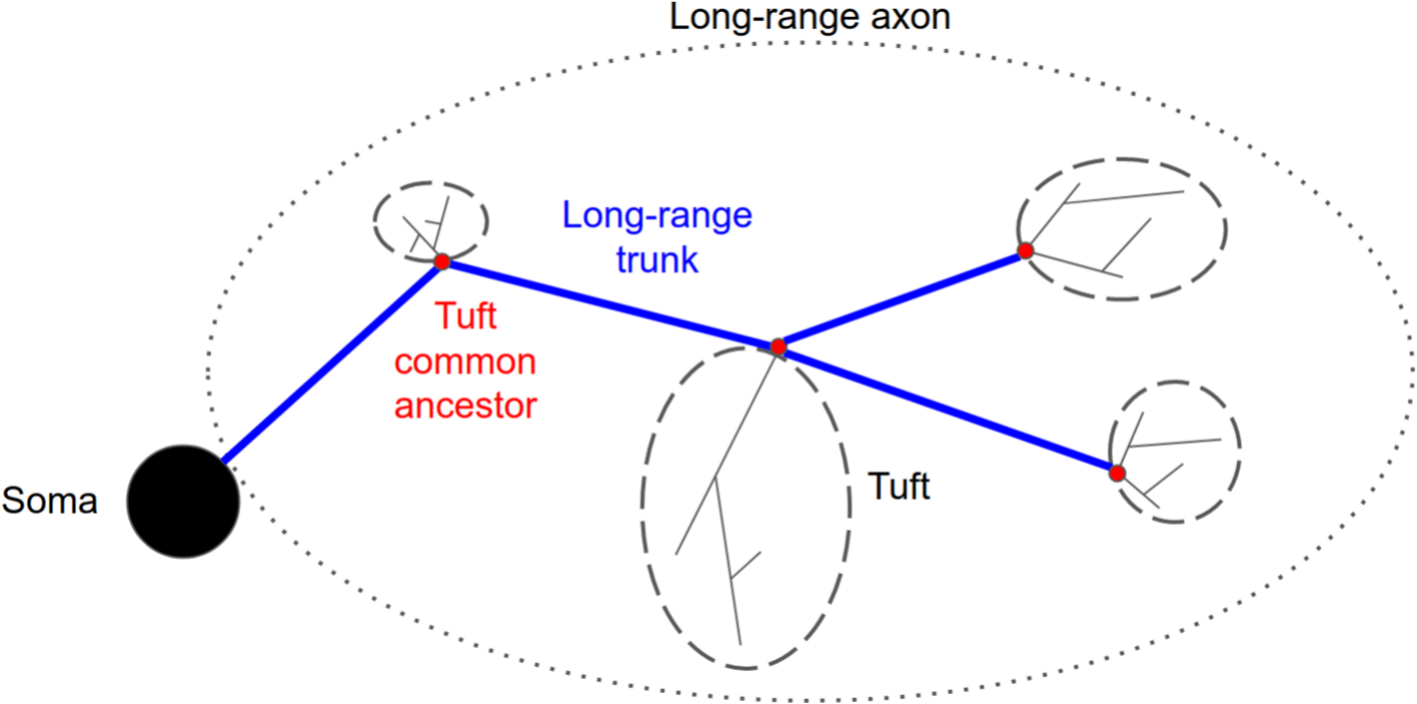
Schematic of long-range axon modeling, including the steps of the long-range trunk, the common ancestors, and the tufts generated at each target point

Long-range axons must target specific brain regions and follow pre-defined pathways, depending on the location of their source and targets (Brodal, 2010; Osten & Margrie, 2013). Various models have been proposed to synthesize artificial neuronal morphologies, each offering different advantages. Some models focus on simulating more realistic axonal growth during brain development, incorporating detailed molecular cues to guide the growth cone (Zubler & Douglas, 2009; Torben-Nielsen & Cuntz, 2014). While these models can reproduce detailed morphologies, they come with significant computational costs and require extensive data, which is difficult to acquire for large-scale circuits. However, mathematical models generate morphologies using mathematical principles and morphological statistics. Although these models require less experimental data, they need manual parameter adjustments based on the type and location of the neurons that are synthesized, therefore they are more difficult to generalize to our case of axonal shapes (Koene et al., 2009; Luczak, 2006; Ascoli et al., 2001; Cuntz et al., 2010).

A recent model successfully integrated the mathematical and statistical approaches into a topological synthesis algorithm (Kanari et al., 2022b). However, this work was limited to the synthesis of dendritic morphologies. Local axons extend within a brain region and similarly to the dendrites they can be simulated taking into account local contextual cues. Long-range axons extend over large distances from the soma and target specific brain regions during development to form functional connections (Dickson, 2002; Sakai & Kaprielian, 2012; Kerstjens et al., 2022; Goodman & Shatz, 1993). Long-range axons must grow taking into account the distance from the soma, as dendrites and local axons do, but also navigate to specific target regions within multiple brain regions (Kollins et al., 2009; Liu et al., 2024; Winter et al., 2023; Zhou et al., 2023). This requirement, along with the need for computational efficiency and realistic local morphometrics, cannot be fully captured by previous models.

In this work, we introduce a novel method for computationally synthesizing long-range axons that reproduce the local morphometrics of reconstructed axons, while also targeting the appropriate brain regions. Our approach utilizes the Steiner Tree Algorithm to generate a skeleton connecting large-scale target regions, followed by a random walk guided by this skeleton to synthesize the primary axonal trunk. Finally, a local-scale model, based on topological synthesis (Kanari et al., 2022b) is applied to generate the terminal branches of the axonal tree. We demonstrate that this method successfully combines large-scale target precision with detailed local-scale axonal synthesis, ensuring accurate targeting of brain regions while preserving realistic local morphometrics.

The organization of the paper is as follows. In Section “Definitions and Modeling Hypothesis”, we briefly define the main concepts used in the following sections, then we describe the method in Section “Methods”. Finally, in Section “Results” we present several results to validate the method and propose an application in a mouse brain atlas.

**Fig. 2:**
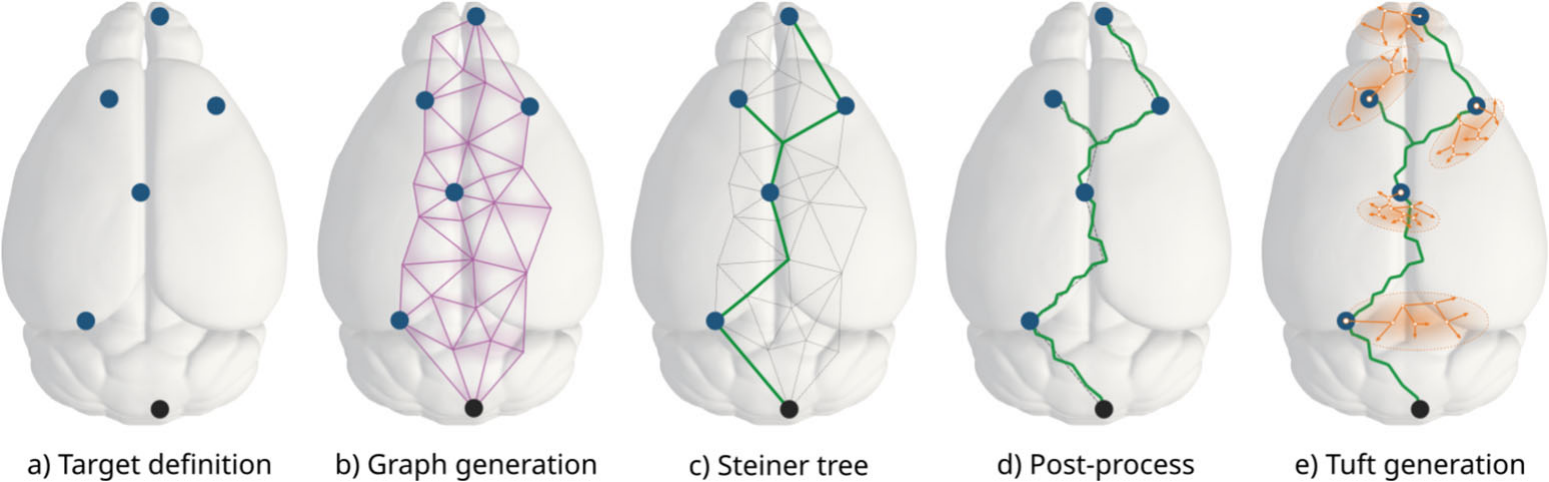
Main steps of the long-range axon synthesis algorithm: define the target points (a), generate a 3D graph containing the source point, the target points, and other intermediate points (b), compute the Steiner Tree that connects the source point to the target points through this network (c), post-process the Steiner Tree solution to make it more realistic (d) and generate one tuft at each target point (e)

## Definitions and Modeling Hypothesis

Axons are complex structures with several components, as detailed in Gibson and Ma (2011). In this work, we divide axons into a long-range trunk, which can span many brain regions, and several local tufts, as shown in Fig. 1. The point connecting a tuft to the long-range trunk is called the *common ancestor* of the tuft. Also, for modeling consistency, the long-range trunk cannot contain any terminals. Terminals are always part of a tuft, so a tuft may contain only one terminal section. The long-range trunk is built so that it connects specific brain regions targeted by the axon. These brain regions are registered in a brain atlas that divides space into voxels and associates data with each of them (brain region ID, local orientation, etc.).

This model is consistent with Gibson and Ma (2011) which states that during the development of the axon, a growth cone extends the axon in the direction of the targeted brain regions to connect to them. During this growth, branching processes occur to connect several brain regions while minimizing the total length of the axonal tree. Then, when the growth cone reaches a targeted brain region, the branch splits into multiple short terminal sections that form synapses with the dendrites of the surrounding neurons. In this work, we aim to create morphologies that are biologically similar to the final state of the developmental process, thus the intermediate states of axonal development are out of scope.

## Methods

In this section, we describe the methodology of the axonal growth. The main steps are summarized in Fig 2. The axon grows in two subsequent steps: first, the trunk is generated based on the Steiner tree (Fig. 2c), then the tufts are created based on a topological synthesis approach (Fig. 2.e).

### Creating Statistical Inputs from Reconstructed Cells

To synthesize realistic morphologies, it is important to calibrate multiple parameters from real reconstructed neuronal morphologies. There are two kinds of parameters to take into account: statistics on local properties of the sections of both the long-range trunk and the tufts, and the global tuft properties that describe their sizes and shapes depending on where they are located.

First, to compute the statistics and properties of the long-range trunk and the tufts, we should define which parts of a reconstructed morphology are considered as tufts. The rest is considered as the long-range trunk. In this work, the tufts are defined based on a simple clustering algorithm: two points separated by a Euclidean clustering distance $d^{\textrm{Euclidean}} \le d^{\textrm{Euclidean}}_{\textrm{max}}$ and a clustering path distance $d^{\textrm{path}} \le d^{\textrm{path}}_{\textrm{max}}$ are considered part of the same tuft. Applying this algorithm to each pair of terminals and merging the pairs with a common terminal defines the tufts. Other clustering algorithms were tested (e.g., do not consider the path or define the tufts according to brain regions), but the one presented here was computationally efficient and gave good results. In the rest of this work, the *clustering distance* stands for the Euclidean clustering distance.

Once the tufts are defined, it is possible to extract the required features from them. For each tuft, its *barcode*, which is a topological signature as described in Kanari et al. (2020), is computed using the Topological Morphology Descriptor (Kanari et al., 2024). Then the following tuft properties are computed:

- the coordinates of the common ancestor,
- the total path length of the tuft,
- the path distance from the soma to the common ancestor,
- the orientation in the local atlas frame of the segment between the common ancestor and the center of mass of the tuft (the local atlas frame has the Y axis towards the pia).

All these properties are then stored in a dataset from which entries will be sampled to synthesize new tufts.

Finally, the mean and standard deviation of the lengths of the segments of the long-range trunk are extracted to make it statistically realistic during synthesis. These properties will be used to post-process the skeleton of the long-range trunk, as the main process described in Section “Connecting SourcePoints to Target Points” only aims at drawing its trajectory, not its actual morphology.

### Building the Long-range Tree

The main trunk of an axon, also referred to as the long-range trunk or long-range tree, is the large-scale part of the axon that connects different brain regions. The synthesis process of the main trunk has 3 main sub-steps:

1. find the source and target point coordinates (step shown in Fig. 2.a, Section “Source and Target Points Placement”);
2. connect the target points (steps shown in Fig. 2.b,c, Section “Connecting Source Points to Target Points”);
3. post-process the resulting tree (step shown in Fig. 2.d, Section “Post-processing”).

#### Source and Target Points Placement

The long-range axon synthesis algorithm starts by collecting the given morphologies from the given cell collection to which the synthesized long-range axons should be grafted. Then the starting points of each axon are computed. These starting points are called the source points of the axons and can either be given explicitly (e.g. to start from an existing local axon) or just start from the soma.

The next step is to associate a *source population* to each long-range axon. These source populations represent groups of neurons that participate in projections to a given set of target brain regions. Source populations can be arbitrarily associated with each axon or computed using a brain atlas, such as Wang et al. (2020). In the latter case, the brain region of the atlas in which the cell is located is computed, then a source population is randomly picked among the ones associated with this brain region (as multiple source populations can be associated with a given brain region to represent the diversity of projection classes). The probabilities of these source populations are given in the input *source population matrix*, which can be derived from connectomics results or from large reconstructed morphology datasets (see Petkantchin et al. (2024) for an application of this work).

After that, the target populations of each axon are randomly picked among the ones possible for the associated source population, each target population possibly being associated with several brain regions. The probabilities of each target population depending on a source population are given in a *projection probability matrix*. Once the target populations have been chosen, a voxel is randomly chosen in the brain region associated with each target population. Then a random shift is applied inside this voxel and the resulting location is called a *target point* of the axon.

#### Connecting Source Points to Target Points

As described by Cuntz et al. (2010), the Ramón y Cajal (1911)’s hypothesis about wiring optimization leads to near-optimal trees regarding the minimization of the total path length of the morphology. In graph theory, the problem of connecting a set of terminal points (which are the target points described in Section “Source and Target Points Placement”) in Euclidean space while minimizing the total length of the graph is known as the Euclidean Steiner Tree Problem. This problem is known to be NP-hard (Karp, 1972), which is an issue when generating large axon structures in a reasonable amount of time. Also, in this problem, it is supposed that the cost function is uniform in space, which is not the case in our problem, as axons tend to follow axonal projection tracts for example. For these reasons, we use an approximation to connect the set of terminal points. As a first step, we transform the Euclidean problem into a graph problem by creating a 3D network connecting all the terminal points with some intermediate points. Then, the edge weights are manipulated to model different constraints (especially to decrease the weight of edges located in the projection tracts). Finally, the Steiner Tree is solved in this graph using the approximation described by Hegde et al. (2015).

The graph generation is divided into several parts. First, the terminal points are collected. Then, several types of intermediate points are added. For the first type, each segment between the source point and the target points is evenly split into sub-segments to create $N_{\textrm{intermediate}}$ new points. These splitting points are used to refine the future graph and to ensure it is possible to connect the terminal points using non-direct paths. Then $N_{\textrm{random}}$ random points are added in the bounding box of the previous points such that that there is no other point within a $r_{random}$ radius. These random points are also used to refine the future graph and to ensure that the Steiner Tree algorithm can find the paths that appear longer in Euclidean space but have smaller weights because they go through preferred areas (e.g. projection tracts, see Section “Preferred Region Modeling”). Then Voronoï points are computed between all the previous points (the Voronoï tesselation is the dual of the Delaunay tesselation, so the Voronoï points are the centers of the circumspheres of the Delaunay tetrahedrons) and added to the set (the ones that are too far from the bounding box are discarded). These Voronoï points allow us to reduce the empty areas and ensure that the bifurcation angles are not too small, which is more realistic. Finally, close points are merged to reduce the size of the point set and to avoid too small segments. This process greatly increases the size of the set of points used to compute the Steiner Tree but gives a lot of possible paths to connect the target points and thus can give relevant solution paths.

Once the set of points is created, a Delaunay triangulation is performed to create the edges of the graph. Then the edges of this graph are weighted according to Eq. 1, where the edge *i* connects the points *A* and *B*, $l_i$ is the length of the edge *i*, $o_i$ depends on the orientation of the edge *i*, $\zeta _i$ depends on the depths of the edge *i* in the atlas and $\gamma _i$ depends on the space area in which the edge *i* is located. The first term of the weight is required since the total length should be as close to the minimum as possible to build a relevant tree. The second term is used to reduce the probability of transverse sections, which are less common. The third term is used to favor paths that follow the curvature of the atlas layers. The last term is required to favor specific areas (e.g. projection tracts).

1 $$\begin{aligned} w_{i}&= l_{i} \times o_{i} \times \zeta _{i} \times \gamma _{i} \end{aligned}$$

Equation 2 defines how each term can be computed. In this equation, *S* is the center of the soma, *C* is the middle of the segment *AB*, $\vec {r}$ is a location in space, $\alpha _{o}$, $\alpha _{\zeta }$ and $\alpha _{\gamma }$ are positive exponents, $\lambda _{o}$, $\lambda _{\zeta }$ and $\lambda _{\gamma }$ are amplitude coefficients, $y(\vec {r})$ is the depth field associating an atlas depth to each location $\vec {r}$ in space and $\gamma (\vec {r})$ is an attraction field that represents the affinity of the axons for each location $\vec {r}$ in space.

2 $$\begin{aligned} \left\{ \begin{array}{ll} l_i & = ||\overrightarrow{AB}|| \\ o_i & = 1 + \lambda _o \times \left( \sin ( \angle ( \overrightarrow{AB} , \overrightarrow{SC} ) ) \right) ^{\alpha _o} \\ \zeta _i & = 1 + \lambda _{\zeta } \times \left( \dfrac{ | y(\vec {r}_A) - y(\vec {r}_B) | }{ || \overrightarrow{AB} || } \right) ^{\alpha _{\zeta }} \\ \gamma _{i} & = 1 + \lambda _{\gamma } \times \left( 1 - \exp { \dfrac{- \left( \gamma ( \vec {r}_A ) + \gamma ( \vec {r}_B ) \right) }{2 \alpha _{\gamma }}} \right) \end{array} \right. \end{aligned}$$

After these steps, the Steiner Tree is computed on the resulting graph to select which edges are part of the final solution (more details about the Steiner Tree algorithm are presented in Appendix F). These edges are collected to build the long-range trunk skeleton. Then, this skeleton will be refined to make small-scale morphometrics more realistic. A simple example of the long-range trunk skeleton building process is shown in Fig. 10.

#### Preferred Region Modeling

As described in Thiebaut de Schotten et al. (2011), the main trunks of the axons usually do not go straight to their targets. In contrast, they tend to use close trajectories in brain regions called axonal projection tracts. Equation 2 presented how the weights of each edge of the graph are assigned before computing the Steiner Tree. In this equation, the last term allows us to consider a set of attractors at arbitrary locations which generate a global attraction field. This attraction field can be used to guide the main trunks of the axons to make them pass through specific regions in the brain, such as the experimentally observed projection tracts (Wang et al., 2020). The right part of Fig. 10 presents the same example as in its left part except that one attractor was added in the upper left corner. In this figure, edges are colored by the linear densities of their weights, where blue-colored edges have small weights and are thus preferred by the Steiner Tree algorithm, while the red-colored edges have high weights and are thus less likely to be selected by the Steiner Tree algorithm. As one can see, edges close to the attractor have smaller weights than edges far from the attractor. As a result, the optimal solution selected by the Steiner Tree algorithm, in this case, is very different from the one without any attractor: the first part of the main trunk passes close to the attractor, and then goes to the closest target, and then connects the furthest target. This simple example demonstrates that it is possible to alter the main trunk shape by defining attractors in space.

In the context of a synthesized axon, multiple attractors may be needed to represent the relevant attraction field. To test and validate the attraction mechanism, an axon was synthesized such that it should be very close to a given reconstructed axon (Fig. 11a to f). In that case, the target points were defined as the common ancestor of each clustered tuft, as described in Section “Mimicking Biological Reconstruc-tions ”. The attractors were built using a new dummy brain region that was added to the atlas composed of all voxels intersected by the reconstructed axon. Then one attractor was created at the centers of a set of voxels of this dummy region. These voxels were randomly chosen such that the distance between two chosen voxels is greater than a given distance. When there is no attractor (Fig. 11a) the main axon trunk goes straight to the target, while when there are enough attractors (Fig. 11b and c) the trunk follows them properly. Nevertheless, a minimum number of attractors is required to ensure good results. In Figs. 11d and e, the attractors are about $300 \mu m$ and $500 \mu m$ apart from each other, respectively, and the trunk deviates from the expected path. And if the attractors are far from each other, for example, $750 \mu m$ apart from each other (Fig. 11f), then the Steiner Tree algorithm ignores the attractors and the trunk goes straight to the target. This example demonstrates that this method can follow complex paths in space when required data are available but that a minimum amount of data is required to provide enough data to the algorithm (i.e. create enough attractors).

#### Post-processing

The previous Section “Connecting Source Points to TargetPoints” described how the long-range trunk skeleton is built to connect the proper brain regions. However, the resulting tree needs post-processing to ensure that its local morphometrics are statistically consistent with the reconstructed axons. This post-process consists of performing a correlated random walk guided by the intermediate points of the Steiner Tree. This random walk must be calibrated so that the result satisfies the local morphometrics extracted in Section “CreatingStatistical Inputs from Reconstructed Cells”.

An important feature for the subsequent growth process of the tufts is to keep immovable the initial target points that will be used as root points to grow the tufts (these root points will thus become the common ancestors of the synthesized tufts). For this reason, the steps described in this section all preserve these points in the morphology. Furthermore, the bifurcation points are also kept immovable for simplicity.

For each tree, a set of long-range trunk statistics is randomly selected among the ones extracted in Section “Crea-ating Statistical Inputs from Reconstructed Cells”. Then a guided correlated random walk is performed between each consecutive immovable point in the tree, whether they are the source point, a bifurcation point, or a target point. The inputs of this random walk are thus a start point, an end point which is the global target to reach, and a set of intermediate points which are intermediate targets close to which the random walk must pass. The direction of each step of this random walk is defined by Eq. 3, where $\vec {u}_i$ is the unit vector pointing in the direction of the current step *i* and which is equal to a weighted sum of several unit vectors: $\vec {u}_{ep}$ is the unit vector pointing to the endpoint, $\vec {u}_{nit}$ is the unit vector pointing to the next intermediate point, $\vec {u}_{hist}$ is unit vector computed from the history of the previous steps and $\vec {u}_{rand}$ is a random unit vector. The $\beta _{i, ep}$, $\beta _{i, nit}$, $\beta _{i, hist}$ and $\beta _{i, rand}$ are the weights applied to each associated term. These weights depend on the current step.

3 $$\begin{aligned} \vec {u}_i&= \beta _{i, ep} \vec {u}_{ep} + \beta _{i, nit} \vec {u}_{nit} + \beta _{i, hist} \vec {u}_{hist} + \beta _{i, rand} \vec {u}_{rand} \end{aligned}$$

At each step of the random walk, the next intermediate target is reassessed to ensure the random walk goes smoothly from one to another. To do this, the distance to the current next intermediate target is evaluated and compared with the value of the previous step. If this distance increased, then the current intermediate target is considered as reached and the next one becomes the new intermediate target. The intermediate target is also considered as reached if the distance is smaller than the mean step length multiplied by a given coefficient (equal to 1 in this work). This process ensures that the random walk follows the long-range trunk skeleton as defined in Section “Connecting Source Points to Target Points”. Nevertheless, the weight coefficients have to be chosen carefully to avoid unexpected behavior. More specifically, $\beta _{i, rand}$ should remain smaller than the sum of the other weights to avoid that the random walk deviates too much from the next intermediate target direction.

After these post-processing steps, the long-range trunk is statistically close to the reconstructed ones and is thus ready to be used to grow the tufts from it.

### Tufts

This section presents the method used to create the tufts and graft them to the long-range trunk (shown in Fig. 2d). For a given tuft, the general process consists of synthesizing a new tuft based on the properties of a reconstructed tuft. This reconstructed tuft is chosen as a template from a set of tufts according to a given weight. This weight is computed for each target population, as defined in Section “Source and TargetPoints Placement”, from a set of properties that should be as close as possible to what is needed for this newly synthesized tuft. These properties can be based on the morphometrics of the tuft or contextual properties. For example, simple morphometrics can be the path length from the soma ($d^{path}_i$) or the total path length of the tuft. A contextual property can be the brain region in which the tuft is located or the distance of the common ancestor of the tuft from the pia.

For example, using the path distance from the soma to the initial point and the expected total path length of the tuft, a probability can be calculated for each reconstructed tuft that may be chosen as a template. For each potential template *j*, an affinity is computed as:

4 $$\begin{aligned} A_j&= Al_j \times Ad_j \nonumber \\&= \mathcal {N}(0, \sigma _l^2)(\overline{l_i} - l_j) \times \mathcal {N}(0, \sigma _d^2)(d^{path}_i - d^{path}_j) \end{aligned}$$

where $A_j$ is the affinity of the template *j*, $Al_j$ is a component of the affinity associated with its total path length, $Ad_j$ is the affinity associated with its distance from the soma, $\mathcal {N}$ is a normal distribution, $\sigma _l$ is the given standard deviation for the expected total path length of the tuft and $\sigma _d$ is the given standard deviation for the path distance from the soma. Then the probability $P_j$ to choose a template *j* is computed as follows:

5 $$\begin{aligned} P_j&= \frac{A_j}{\sum _k A_k} \end{aligned}$$

**Fig. 3:**
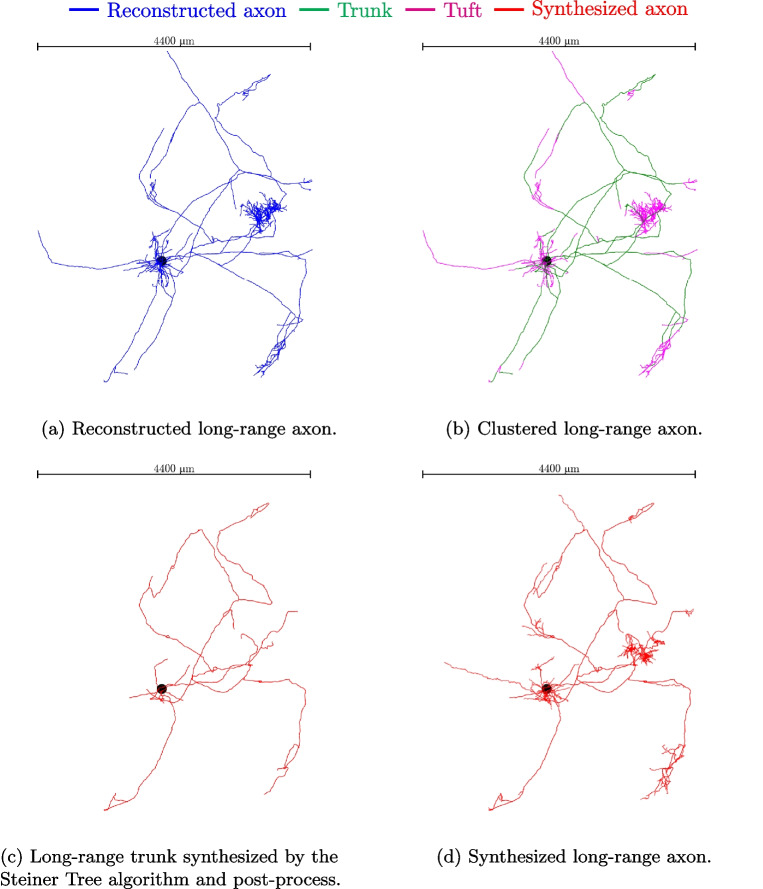
Main steps of the long-range axon mimic algorithm, including the steps of clustering the input morphologies (Section “Creating Statistical Inputs from Reconstructed Cells”), synthesizing the main trunk (Section “Building the Long-range Tree”) and synthesizing the tufts (Section “Tufts”). The black spheres show the soma positions but the radius was increased to make it visible

Once a template tuft is chosen, a new tuft is synthesized using the NeuroTS algorithm (Kanari et al., 2022a). This algorithm aims at synthesizing topology-guided neuronal morphologies by performing a constrained random walk. It uses topological representations of the morphologies called *barcodes*, as defined in Kanari et al. (2024), which represents the length to synthesize before creating a bifurcation in the tree, and a few statistical properties such as the randomness amplitude and the mean and standard deviation of the step size of the random walk. The barcode given as input is the one of the template tuft, so the total length of the synthesized tuft is expected to be close to the one needed. In this work, all synthesized tufts use the same set of parameters and distributions in the NeuroTS algorithm, but it is possible to use specific ones for each tuft to refine the results.

Finally, the synthesized tuft is grafted onto the target point which becomes its common ancestor.

## Results

In this section, we present the validation of the computational synthesis by comparing computationally generated axons to reconstructed morphologies. We first establish the method’s reliability by demonstrating its ability to precisely mimic reconstructed axons. A critical component of our analysis focuses on examining the impact of cluster size, primarily determined by the Euclidean clustering distance. To illustrate the method’s practical application, we present a case study that leverages reconstructed morphologies to identify axon target points and use atlas data to follow the projection tracts within a mouse brain.

### Mimicking Biological Reconstructions

To verify that the method presented in Section “Methods” can build realistic long-range axons, the capacity to synthesize axons similar to reconstructed ones is evaluated.

The main steps of the so-called *mimic* algorithm, when used to mimic a reconstructed morphology, are summarized in Fig. 3. In the first step (Fig. 3a), a given reconstructed axon is clustered to select its tufts and reduce them to the common ancestors of their clusters, as described in Section “Creating Statistical Inputs from Reconstructed Cells”. Figure 3b shows the biological reconstruction after the tuft clustering: tuft sections are represented in magenta while the trunk is represented in green. Then, a graph is created as described in Section “Connecting Source Points to Target Points”. In this case, the preferred regions as described in Section “Preferred Region Modeling” are enabled. These preferred regions are constructed along the reconstructed morphology, as presented in Section “Preferred Region Modeling”. Then we use the Steiner Tree algorithm (Section “ConnectingSource Points to Target Points ”) to build the long-range trunk that will use the common ancestors of the clustered tufts as target points. This synthesized trunk is then post-processed as described in Section “Post-processing”. Figure 3c presents the synthesized trunk after this post-process. Finally, the tufts are synthesized as described in Section “Tufts”, based on the properties of the clustered tufts of the reconstructed axon. Figure 3d shows the final result of the synthesis process.

These figures show that the global shape of the synthesized long-range axon is very close to the one of the initial reconstructed morphology. The long-range trunk goes to the proper target areas, and the tufts have similar sizes and orientations, so the target regions are properly innervated. Nevertheless, the branches of the long-range trunk are not identical. This is expected because the intermediate points given to the Steiner Tree algorithm are not only based on biological features and are partially random, so the result can not be the same as the reconstructed morphology. Also, one can see that the parallel trunks seen in Fig. 3a (e.g., in the lower-left part of the morphology) are replaced by a single trunk in Fig. 3d, this is because the Steiner Tree Algorithm tries to minimize the total length of the trunk, so the parallel trunks can only appear if there is a forbidden area between them. This specific feature is not covered in this work as it is hard to get such data and because we suppose that the trunks have a small contribution to the connection network compared to the tufts.

In addition, Fig. 9 shows that most local morphometrics are consistent between the synthesized and reconstructed axons. Some morphometrics are not consistent though, especially the mean section lengths and, to a lesser extent, the inter-segment angles. The section length is expected to be different because the bifurcations in the trunk have no reason to be located at the same places.

**Fig. 4:**
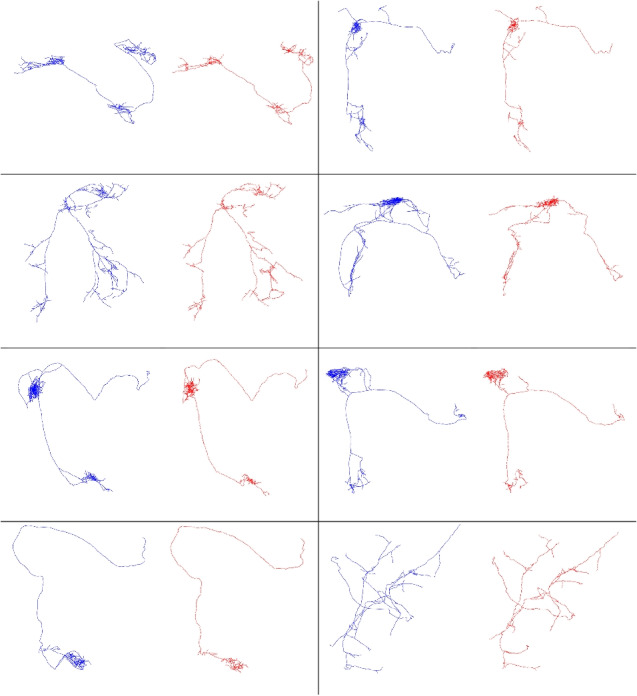
Examples of synthesized long-range axons (red) and the respective reconstructed axons (blue). These results demonstrate the ability of the algorithm to approximate a variety of different shapes

Figure 4 shows several examples of synthesized axons that mimic reconstructed ones. The clustering distance is $100~\mu$m for all of them. Again, the results show good accuracy.

**Fig. 5:**
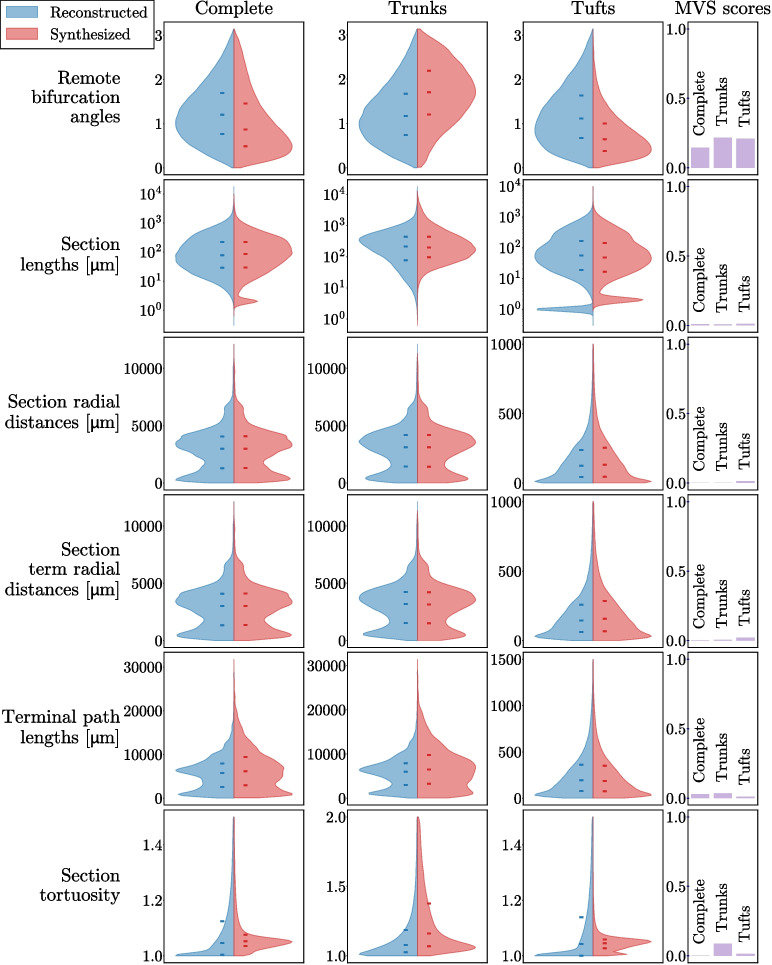
Distribution of morphological features across 1076 reconstructed (blue) and synthesized (red) axonal complete morphologies (left column), trunk parts of the morphologies (middle column), and tuft parts of the morphologies (right column). Median, first, and third quartiles are shown on the distributions (hyphens). The MVS statistical score is shown in the last column for each type of morphology

In addition, to verify that the synthesized morphologies are statistically similar to the biological reconstructions, we mimicked 1076 morphologies of the MouseLight project (Winnubst et al., 2019). These morphologies, extracted from adult mice ($\text {age} > 8 ~\text {weeks}$), originate and target various brain regions. We compared the distributions of morphometrical features across the reconstructed and synthesized populations. These distributions for the complete morphologies, the trunks alone and the tufts alone are plotted in Fig. 5. The morphometrics used are defined in the Petilla terminology (Ascoli et al., 2008; Kanari et al., 2022b) and are briefly described in Appendix E. We also plotted the Maximum Visible Spread (MVS) score, defined in Kanari et al. (2022b), for the complete morphologies, the trunks alone and the tufts alone in Fig. 5. This score gives an indication of the similarity between two statistical distributions, by computing the absolute difference between the medians divided by an estimate of the overall visible spread of the distributions. This score is minimal at 0 when the medians coincide and the distributions are very close to each other; the closer it gets to 1, the less similar the distributions are. The morphometrics of the reconstructed and synthesized axons match very closely at a population level, with the worst MVS of 0.12, 0.20, and 0.16 for the remote bifurcation angles of the complete, trunk and tuft morphologies respectively. The cause of this difference in remote bifurcation angles is still unclear and will require more work. It could be due to a fundamental difference between the axonal tuft and dendrite development that lead to small deviations, but the data on this aspect are still sparse.

**Fig. 6:**
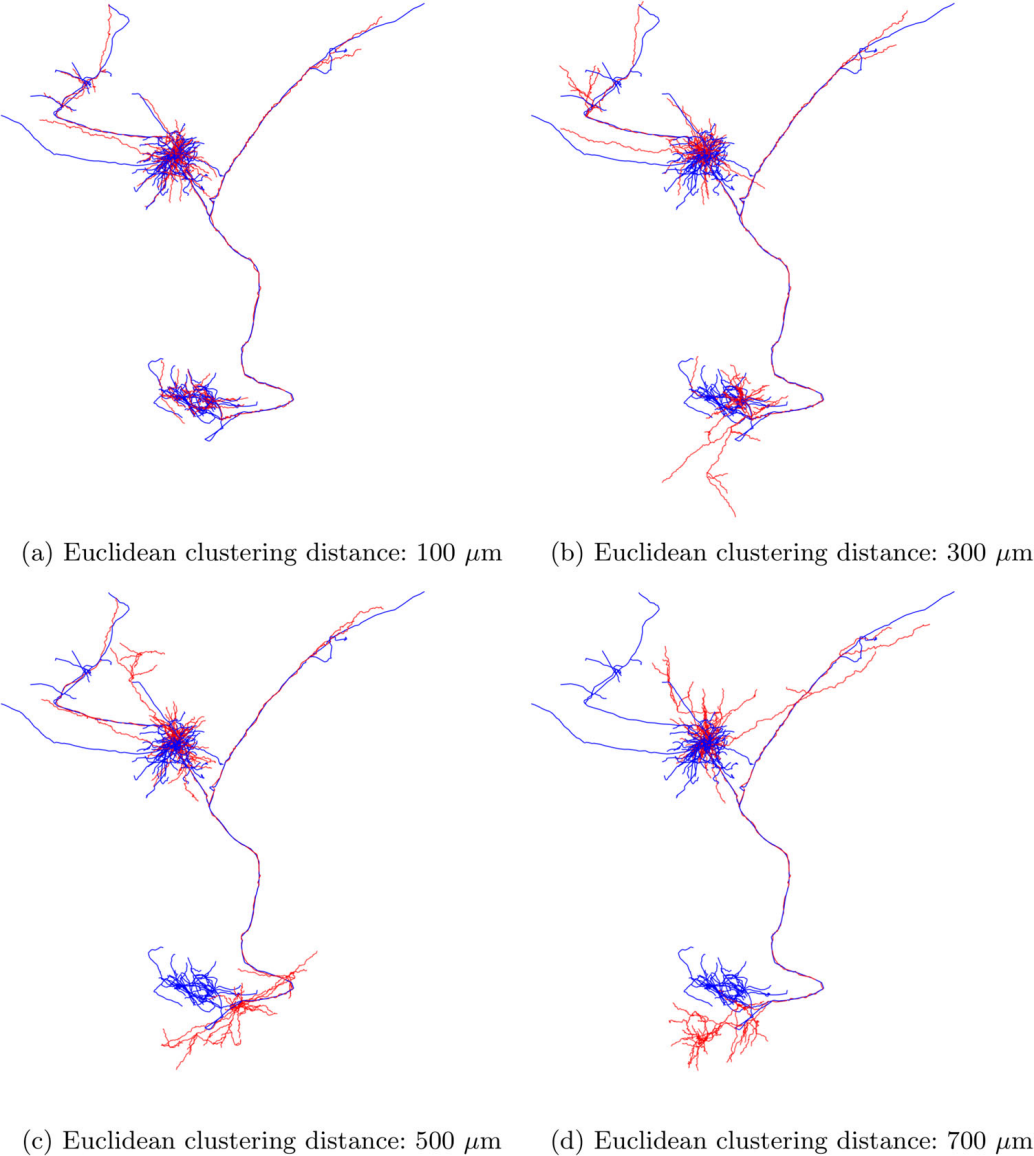
Parameter selection for clustering distance. Synthesized long-range axons (red) for varying Euclidean clustering distances compared to reconstructed axons (blue). Smaller clustering distance approximates the original axons with higher accuracy. In these figures, the soma is located inside the large tuft in the upper part of the morphology

### Clustering Distance Effect

The Euclidean clustering distances used to define the tufts in the reconstructed morphologies is a crucial parameter when aiming to achieve results that closely match the reconstructed morphology. Small clustering distances tend to yield more detailed and accurate representations. However, in practical applications, using such small clustering distances can be challenging due to data accuracy and depends on how the target points are defined. As a result, it becomes necessary to find a compromise between the desired accuracy and the practical feasibility. Figure 6 shows the effect of the Euclidean clustering distance (with a path distance equal to $d^{path}_{max} = 3 \times d^{Euclidean}_{max}$) on a given synthesized axon compared to the corresponding reconstructed axon. As can be seen, the synthesized axon is very close to the reconstructed one when the clustering distance is small (Fig. 6a) while it becomes less and less accurate with higher clustering distance values (Fig. 6b to d). Figure 12 shows the normalized *L*1 error of the projection intensity for each Euclidean clustering distance. The projection intensity plotted in this figure is computed as follows: a 3*D* grid is created with a given voxel size, then the intersection of the axon and each voxel is computed for both the reconstructed and the synthesized axons. Then the normalized *L*1 error between the voxels of the two previous results is computed with Eq. 6 where $r_i$ is the value of the voxel *i* of the reconstructed result and $s_i$ is the value of the voxel *i* of the synthesized result.

6 $$\begin{aligned} L_1 = \dfrac{\sum || r_i - s_i ||}{\sum r_i + \sum s_i} \end{aligned}$$

The process is repeated for multiple voxel sizes in order to show which scales are properly synthesized and which are not. The result shows that for a clustering distance equal to $100~\mu$m the synthesized axon has very similar intersection lengths as the reconstructed axon as long as the voxel size is bigger than $250~\mu$m, while the error increases fast for lower voxel sizes. This means that the spatial structures bigger than this scale are accurately synthesized while the smaller structures are less accurate, which is expected since the synthesized axon is only supposed to be similar to the reconstructed one but not strictly equal, especially in the tuft areas. For bigger clustering distances, the size of accurately synthesized structures is much bigger.

**Fig. 7:**
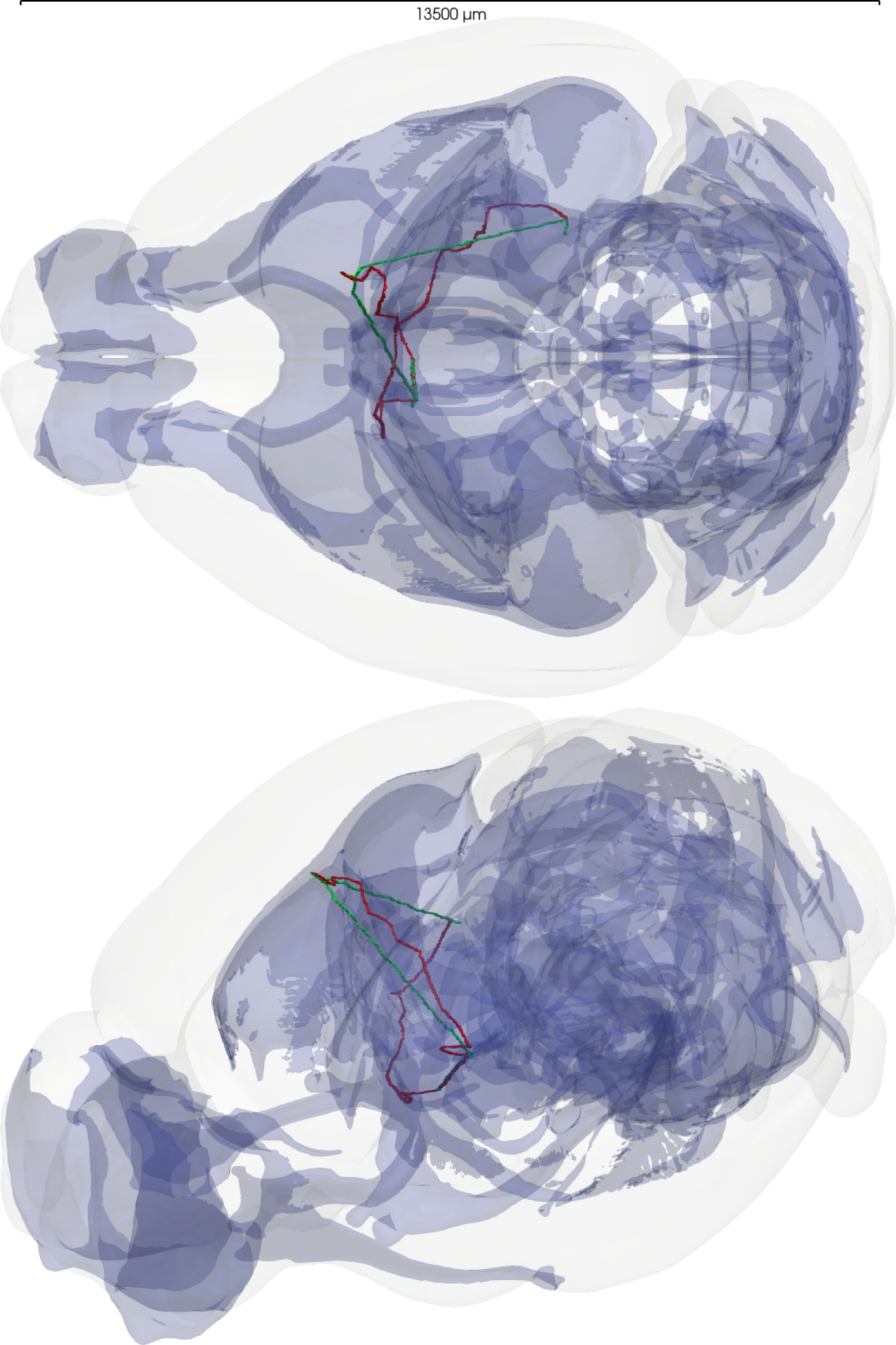
Comparison of two synthesized long-range axons, whose somata are located in the Primary Motor area, within the brain Atlas. The preferred region feature is disabled for the green one while it is enabled on the projection tracts for the red one. The two sub-figures show the same result from different points of view (top view in the upper figure and front left oblique view in the lower figure)

### Application: Connecting Mouse Brain Regions

All previous tests have shown that the presented model is capable of accurately synthesizing long-range axons in many different situations. This last section presents a possible use case in which reconstructed morphologies are used to define the target point locations in the brain atlas. In this example, 44 morphologies from Winnubst et al. (2019) with somata located in layers 5 and 6 of the Primary Motor Area (MOp5 and MOp6 regions) were clustered to define trunks and tufts. Random points were then selected around these tuft locations using a normal distribution with a standard deviation equal to $200~\mu$m, while ensuring that the selected points remained in the same brain region as the initial tuft point. The properties of the tufts were directly used for picking the barcodes during the synthesis of the new tufts.

#### Projection Tracts as Preferred Regions

Using this setup, we first checked the effect of the preferred region feature. Here, all the brain regions considered as projection tracts in the CCFv3 atlas (Wang et al., 2020) are used as preferred regions. In this context, two long-range axons were synthesized using the same source and target points, the only difference being whether the preferred regions were enabled or not. As can be seen in Fig. 7, the two axons are very different: when the preferred regions are disabled, the main trunk goes straight to the target points. When preferred regions are enabled the trunk follows the projection tracts when possible and then goes to the target points. This example shows that the model can follow realistic projection tracts in the brain, which is a key feature for realistic long-range axon synthesis.

**Fig. 8:**
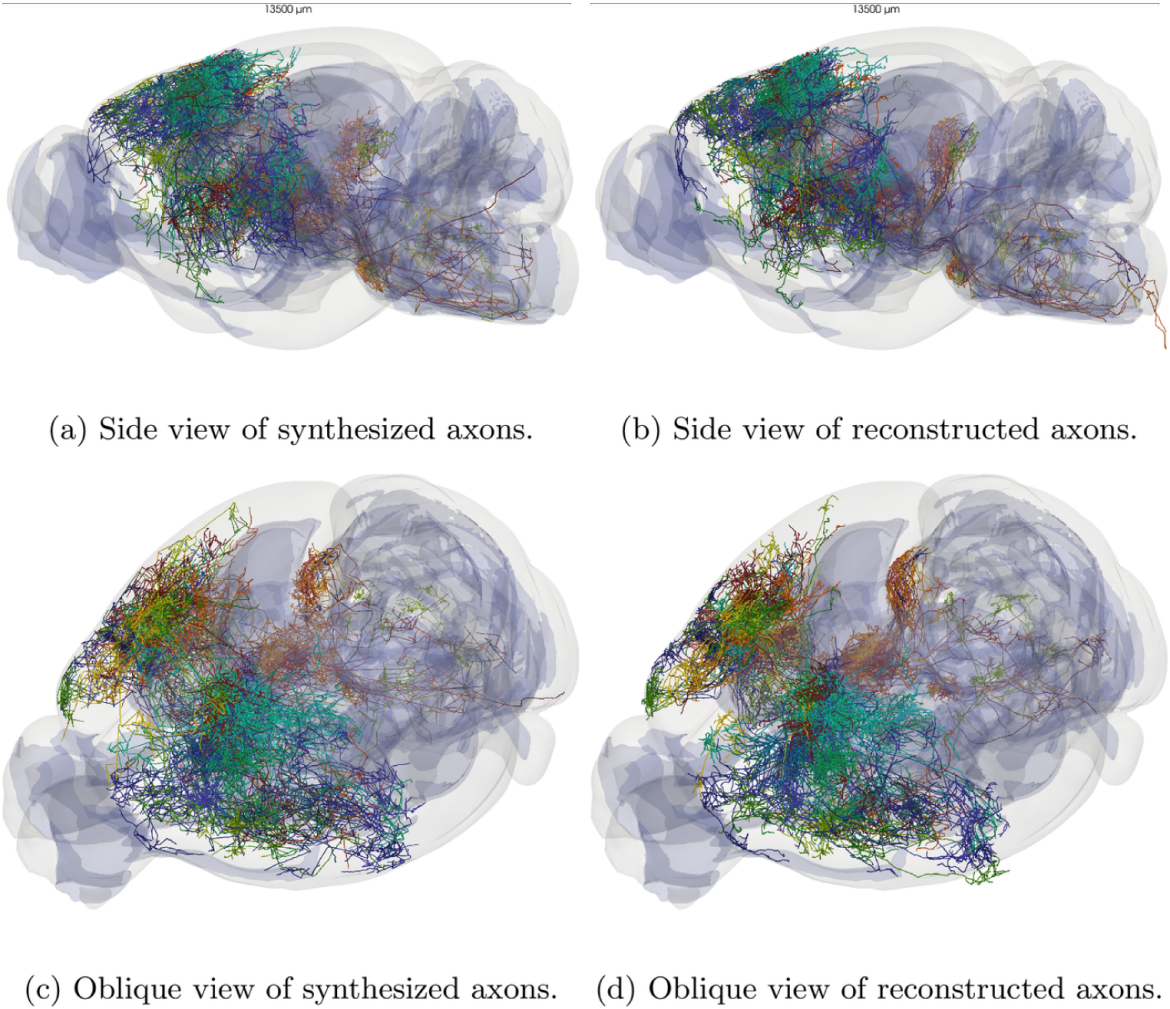
Example of 44 long-range axons synthesized such that follow the projection tracts (blue region) (a and c) compared to the respective 44 reconstructed morphologies (b and d). Each axon has a different color

#### Boundary Constraints

This application aims at synthesizing long-range axons in a specific spatial context, namely that of a mouse brain. In this context, an important feature is to ensure that the synthesized morphologies do not go outside of the brain. This is done by applying simple boundary constraints. The first constraint only applies to the long-range trunk since it is applied during the graph creation step described in Section “Connecting Source Points to Target Points ”. During this step, all the points of the graph located outside the brain are discarded before computing the Steiner Tree. This ensures that the long-range trunk does not go outside the brain. The second constraint only applies to the tufts, since it is applied during the tuft generation step described in Section “Tufts”. First, the space is divided into a 3D grid, the same as the one containing the atlas brain regions. Then for each voxel of this grid, the vector from the center of the voxel to the closest boundary point is calculated, so we obtain a 3D vector field, called the *boundary field*, each vector representing the distance and direction to the closest boundary point. Finally, the vectors located outside the brain are reversed and resized to a small length ($1e^{-3} \mu m$ in this work) to make the space outside the brain repulsive. After this process, the growth of the tufts is modified so that at each step of the random walk the distance and direction of the current point to the closest boundary are extracted from the previous boundary field. This distance is then refined to consider the position inside the voxel and used to calculate an attenuation vector using the following equation:

7 $$\begin{aligned} \vec {a}_i = \dfrac{\vec {b}_i}{||\vec {b}_i||} \times \exp \left( - \alpha _b \times ||\vec {b}_i|| \right) \end{aligned}$$

where $\vec {a}_i$ is the attenuation vector for the point *i*, $\vec {b}_i$ is the vector from the boundary field at the location of the current point *i* and $\alpha _b$ is a scaling coefficient. The refined distance is calculated by a simple linear extrapolation, considering that the attenuation field keeps the same direction inside a voxel. So, the vector from the current point to the center of the voxel can just be projected on the vector associated with the voxel and added to it to refine the distance to the boundary.

**Table 1:** MVS scores of the morphometrics distributions computed on the 44 morphologies synthesized with and without the boundary constraints enabled. These MVS scores are very close to 0 so the morphometrics distributions are almost identical whether the boundary constraint is enabled or not

Morphometric name	MVS score
Remote bifurcation angles	0.009667
Section lengths	0.000772
Section radial distances	0.002622
Section term radial distances	0.002159
Terminal path lengths	0.004756
Section tortuosity	0.001269

Finally, this attenuation vector is added to the direction of the current step of the random walk to reduce the component in the direction of the boundary. This process will reduce this component more and more so that it cannot cross the boundary.

#### Multiple Axon Synthesis

Using the same setup, 44 long-range axons were synthesized. The model is again configured to follow the projection tract regions of the CCFv3 atlas (Wang et al., 2020). The main parameters given to the model are presented in Table 2. Figure 8 shows that the long-range axons follow the projection tracts and properly innervate the same brain regions as the reconstructed morphologies. In this result, we notice that the synthesized axons do not always follow the same tracts as the reconstructed morphologies. This is because all the tracts were considered equally attractive in this application for simplicity, but this is not the case in reality and this approach should be refined for a real application.

Finally, we compared a set of morphometrics when boundary constraints were enabled or disabled to ensure that this process did not have a sensitive impact on the local structure of the morphologies. Table 1 presents the MVS scores for several morphometrics of the morphologies synthesized without the constraint compared to those calculated with the constraint. Most of these morphometrics are very similar (MVS scores are all very close to 0). Note that tortuosity and radial distances are expected to be different since the sections close to the boundary are curved when the boundary constraints are enabled.

### Benchmarking

The method presented in the previous sections was developed to be computationally efficient. The algorithm was parallelized at morphology scale using MPI. The implementation relies on the *dask* framework, especially the *dask-mpi* extension. The main inputs are computed using vectorial computation on a global scale, before the tasks are scattered among the workers.

To estimate the computational cost of the algorithm, a test was performed in which 100000 long-range axons were synthesized on a super-computer with 200 tasks. The morphologies started from the Primary Motor Area (MOp) and expanded to multiple other regions in the brain. Parameters were consistent with those used in Section “Multiple Axon Synthesis” and both the preferred regions (for the projection tracts) and boundary features were enabled. In these conditions, the test is completed in 33min 15s using about 4*Go* of memory per task. Note that this time could be reduced by selecting the proper subset of projection tracts for each morphology. Indeed, all the projection tracts were selected as preferred region for each morphology, which results in a very large number of points in the graph on which the Steiner tree is computed. Therefore, the computation time reported in this benchmark should be considered as an upper boundary for real applications. The total length synthesized considering all axons was equal to $1.01 \times 10^3$m, which means that the mean length of the synthesized axons is equal to $1.01 \times 10^4\mu$m. This computational speed enables one to synthesize large neuronal circuits in a reasonable time.

## Discussion

The synthesis of long-range axons is a complex yet essential step in reconstructing complete brain networks. In this work, we introduce a novel algorithm to synthesize long-range axons accurately. The synthesized axons show strong statistical agreement with expected biological properties, including local morphometrics, global targeting, and intermediate trajectories through preferred regions, such as projection tracts. Thus, the presented algorithm enables the generation of a diverse range of axons contributing to the reconstruction of large-scale brain circuits. In addition, the method is computationally efficient, and can thus generate 100000 axons of approximately 1km total length in about half an hour on a cluster with 200 tasks.

The axon synthesis algorithm can be further refined to generate more accurate axonal morphologies and, importantly, to increase variability within computational models of long-range axons. The current version shows sensitivity to the quality of input data, as shown in Section “ClusteringDistance Effect”. As more experimental data becomes available, the algorithm should generalize to capture the global properties of long-range axons to minimize dependency on specific experimental inputs. Additionally, the selection of projection tracts should be guided by the source and target locations of each axon, as outlined in Budd et al. (2010) and Thiebaut de Schotten et al. (2011), while also accounting for the different neuronal classes, as described in Liu et al. (2024).

Finally, it would be important to use population statistics to refine the inputs of the algorithm. Two recent studies, Venkadesh et al. (2023) and Wheeler et al. (2024), present methods to identify specific projection types that could be used to refine the targeting inputs for axons, based on population statistics. For example, target or intermediate points that are used as Steiner Tree inputs could be chosen based on specific sub-region patterns for different projection neuronal types, as observed in experimental data (Venkadesh et al., 2023). This improvement would greatly increase the accuracy of the synthesized neuronal circuit and reduce dependence on the specific input data. In addition, we expect that this refinement would not significantly impact computational performance, with the potential to actually reduce computational cost by reducing the number of nodes in the graph explored by the Steiner Tree algorithm.

Another potential improvement is to modify the Steiner tree algorithm to achieve a balance between cable cost and distance from the source, following an approach similar to the Tree algorithm, as described in Cuntz et al. (2010). Along with optimizing the selection of Steiner parameters, we will also implement an enhanced version of source-target connectivity. These modifications will improve specific morphometrics that currently do not perfectly match experimental data, such as inter-segment angles, bifurcation angles, and branch orders.

In a companion study, Petkantchin et al. (2024), we focus on generalizing the axon targeting from the available biological inputs for each brain region and hemisphere, and adding specialized tuft selection and placement to avoid replicating individual morphologies, thereby more accurately capturing population variability. By adding a clustering process to better differentiate the synthesis inputs, we also capture the source and targets of axons more accurately, improving, in addition, the input positions to the Steiner Tree algorithm. These improvements, alongside the incorporation of additional experimental data, will further enhance the quality and biological relevance of the synthesized axons.

The axon synthesis algorithm enables the generation of large-scale neuronal circuits with biologically realistic connectomes, generating detailed computational brain models. This capability allows for in-silico experiments that are otherwise unfeasible in-vivo, providing a powerful tool for advancing neuroscience research. The integration of synthesized brain regions with electrical models (Ofer et al., 2017; Rama et al., 2018) further enables the study of signal propagation across large neuronal circuits (Markram et al., 2015; Reimann et al., 2024). This step is essential for simulating brain disorders and investigating specific neurological conditions such as autism, epilepsy, Alzheimer’s disease, Parkinsons’s disease, and other neurodegenerative disorders (Belmonte & Bourgeron, 2006; Kaufmann and Moser, 2000; Moolman et al., 2004; Srivastava et al., 2012; Torben-Nielsen & Cuntz, 2014; Koch et al., 2015). While the full validation of such models remains a challenge, this approach holds significant promise for improving early-stage diagnostics and accelerating drug discovery. Moreover, this translational approach offers the potential for personalized medicine, enabling patient-specific drug testing and therapeutic strategies over the long term.

## Information Sharing Statement

All data and scripts used to generate the results presented in this work are available here: https://doi.org/10.5281/zenodo.13843537

The main code used for axon synthesis is available here: https://github.com/BlueBrain/axon-synthesis

## Figures and Tables

**Fig. 9:**
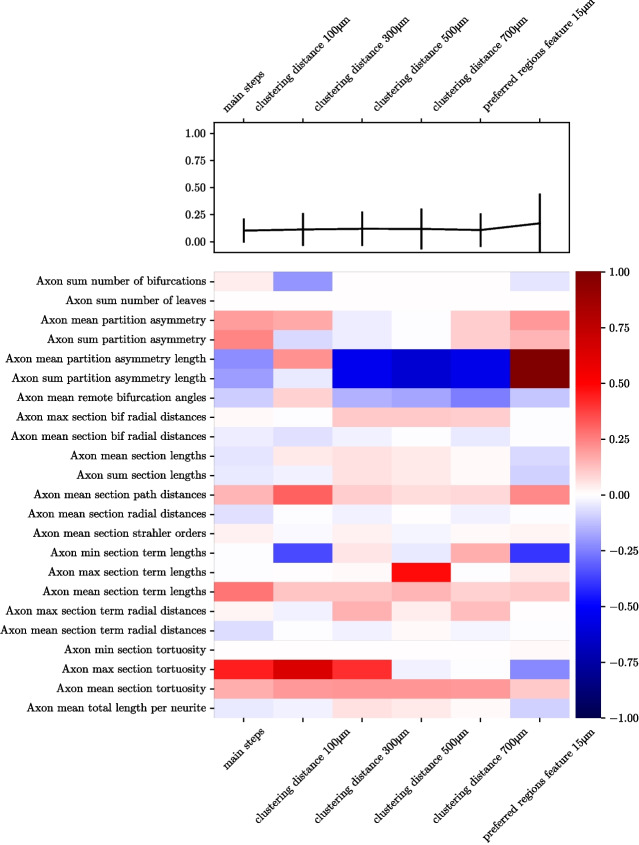
Errors on local morphometrics for different axons presented in this work. White cells mean that the morphometric values for synthesized and reconstructed axons are very close while red/blue cells mean that the synthesized value is much higher/lower. The upper part of the figure shows the global error on all the morphometrics with error bars. These morphometrics are defined in the Petilla terminology (Ascoli et al., 2008) and details about their computation are given in the NeuroM documentation

**Fig. 10:**
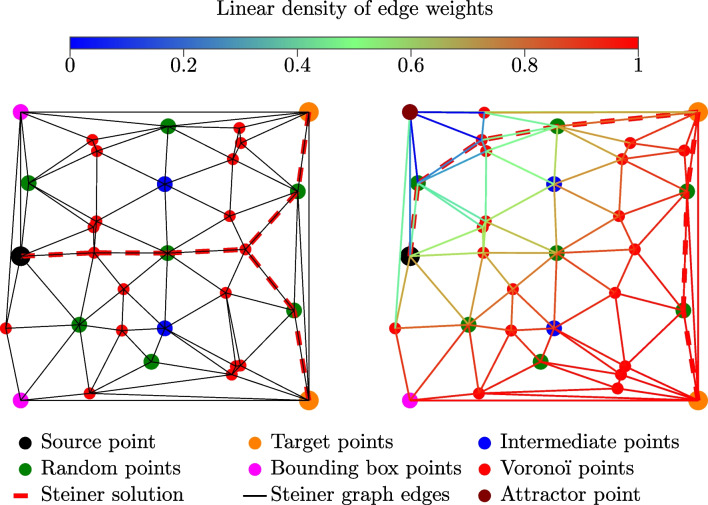
Example of graph creation and Steiner Tree solution on a simple case when there is no attractor (left) and with one attractor in the upper left corner (right). When there is no attractor (left), the optimal path minimizes the total length. When there is an attractor (right), the optimal path is very different because the edge weights are not only related to their length, but also to their distance to the attractor. All edges have the same linear density in the left figure while their linear density is given by their color in the right figure

**Fig. 11:**
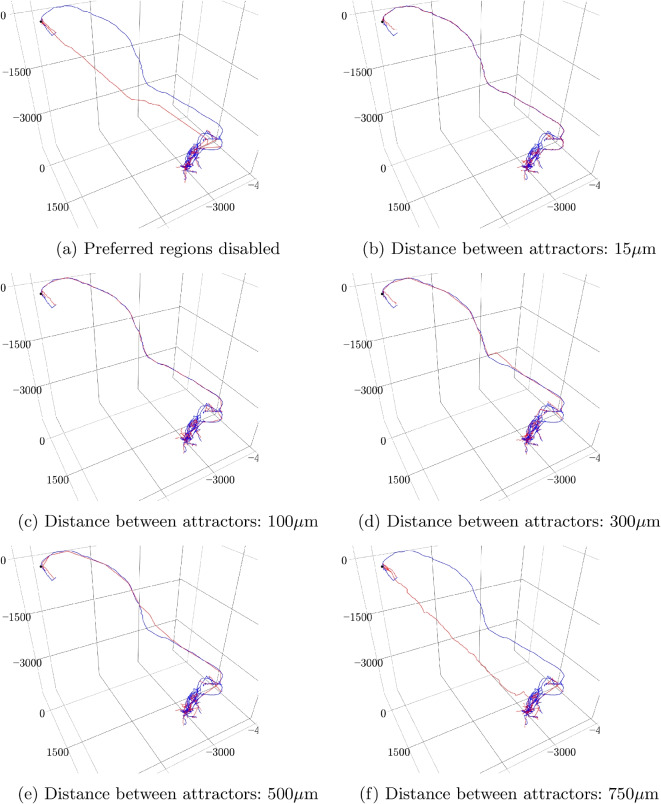
Examples of synthesized long-range axons (red) compared to reconstructed axons (blue) when attractors in the preferred regions are disabled (a) or enabled (b-f). The minimum distance between attractors has an important effect on the result: the smaller the distance, the better the results. At some point, especially in (f), the algorithm is no more able to follow the preferred region because there are not enough attractors to represent it. But of course, the smaller the distance, the longer the computation time, so a compromise must be found. Axis ticks are given in $\mu$m in all subplots. In these figures, the random components of the post-process was set to a low value to highlight the behavior of the Steiner Tree algorithm. The black spheres show the soma positions but the radius was increased to make it visible

**Fig. 12:**
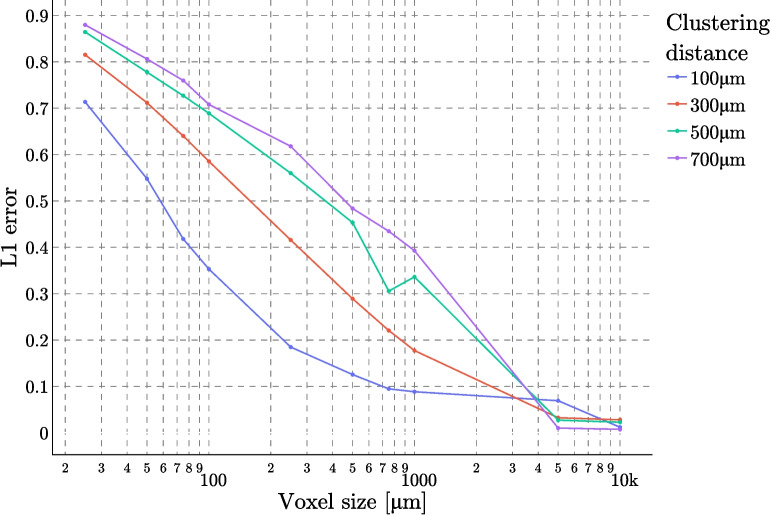
Projection intensity errors for different Euclidean clustering distances (the path clustering distance is set to $d^{path}_{max} = 3 \times d^{Euclidean}_{max}$) for the morphology presented in Fig. 6. This L1 error shows that the smaller the clustering distance the better the results

**Fig. 13:**
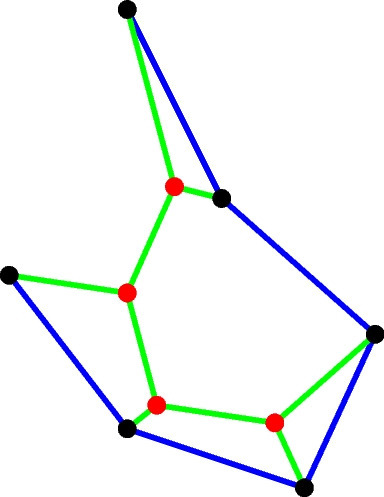
Example of a minimum spanning tree (blue) and a Steiner tree (green) that connect the same set of points (black). The Steiner tree is allowed to create new intermediate points (red), called Steiner points, to reduce the total length of the network

## Data Availability

All data and scripts used to generate the results presented in this work are available here: https://doi.org/10.5281/zenodo.13843537

## Appendix A: Synthesis Parameters

**Table 2:** Input parameters used to generate the long-range axons presented in the different sections of this work

Synthesis case	Synthesis step	Parameter	Value
Morphometrics presented in Section “Mimicking Biological Reconstructions” (Fig. 5)	Clustering	$d^{Euclidean}_{min}$	$300 \mu m$
		$d^{path}_{max}$	$1000 \mu m$
	Graph creation	$\alpha _o$	0.1
		$r_{random}$	$500 \mu m$
		$\lambda _o$	1
		$\alpha _{\zeta }$	0.25
		$\lambda _{\zeta }$	2
		$\alpha _{\gamma }$	100
		$\lambda _{\gamma }$	1
Long-range axons presented in Section “Projection Tracts as Preferred Regions” (Fig. 7)	Clustering	$d^{Euclidean}_{min}$	$200 \mu m$
		$d^{path}_{max}$	$300 \mu m$
	Graph creation	$\alpha _o$	0.1
		$r_{random}$	$500 \mu m$
		$\lambda _o$	1
		$\alpha _{\zeta }$	0.25
		$\lambda _{\zeta }$	2
		$\alpha _{\gamma }$	10
		$\lambda _{\gamma }$	5
Long-range axons presented in Section “Multiple Axon Synthesis” (Fig. 8)	Clustering	$d^{Euclidean}_{min}$	$200 \mu m$
		$d^{path}_{max}$	$300 \mu m$
	Graph creation	$\alpha _o$	2
		$r_{random}$	$500 \mu m$
		$\lambda _o$	2
		$\alpha _{\zeta }$	0.25
		$\lambda _{\zeta }$	2
		$\alpha _{\gamma }$	30
		$\lambda _{\gamma }$	7

## Appendix E: Petilla Terminology

This section briefly describes the morphometrics used in this work (Section “Mimicking Biological Reconstructions”, Fig. 5 and Table 1).

- **Remote bifurcation angles:** The remote bifurcation angle is the angle between two out-going sections in a bifurcation point, defined as the angle between the bifurcation point and the last points in the out-going sections.
- **Section lengths:** The section length is calculated as the sum of the lengths of all the segments that compose the section.
- **Section radial distances:** The radial distance is the Euclidean distance between the end-point point of the section and the center of the soma of the morphology.
- **Terminal path length:** The terminal path length is the sum of the lengths of all the sections on the path from the soma of the morphology to the given terminal.
- **Section tortuosity:** The section tortuosity is defined as the ratio of the length of a section and the Euclidean distance between its end points.

## Appendix F: Euclidean Steiner Tree Problem

The Euclidean Steiner Tree problem is a fundamental optimization problem in network design that generalizes the minimum spanning tree problem. Consider a set of points in a metric space. The goal is to find a tree (a connected graph without cycles) that connects all these points while minimizing the total distance or cost of connections.

What makes the Steiner Tree problem distinct from a minimum spanning tree is that it is allowed to add additional points, called Steiner points, to achieve a shorter total distance. Figure 13 shows the difference between a minimum spanning tree and a Steiner tree in a simple case. These extra points serve as *junction points* or *intermediary nodes* that can reduce the overall cost of the network.

The Steiner Tree problem, especially the Euclidean variant with obstacles that is needed for this work, is NP-hard (Karp, 1972), meaning there is no known polynomial-time algorithm to solve it exactly. However, there are various approximation algorithms and heuristics that work well in practice. In this work, the Euclidean variant was transformed into a graph variant and the method from Hegde et al. (2015) was used to approximate the Steiner Tree solution.

## Appendix G: Morphology References

**Table 3:** IDs of the morphologies used in each part of this work

Section or figure	Morphology reference in the MouseLight dataset
Section “Mimicking Biological Reconstructions” and Fig. 3	AA0001
Figure 4	84991, 84992, 84994, 84995, 84997, 85002, 85006
Figure 5	all morphologies with long-range axons from 84987 to 260149
Figure 6	121514
Figure 8	85066, 85067, 85070, 85079, 85083, 85084, 85086, 85094, 85096, 85097, 85098, 85101, 85102, 85218, 85219, 85221, 85222, 85224, 85225, 121511, 121512, 121514, 121527, 121567, 121648, 121649, 121652, 121674, 121675, 121677, 121678, 121685, 121686, 121729, 121730, 121732, 122028, 122029, 122031, 260064, 260079, 260089, 260110, 260128

All these morphologies are publicly available (see Information Sharing Statement)

## References

[CR1] Adamovich T, Ismatullina V., Chipeeva, N., Zakharov, I., Feklicheva, I., & Malykh, S. (2024). Task-specific topology of brain networks supporting working memory and inhibition. bioRxiv. Pages: 2024.04.05.588287 Section: New Results. 10.1101/2024.04.05.588287 10.1002/hbm.70024 PMC11387957 39258339

[CR2] Ascoli, G. A., Alonso-Nanclares, L., Anderson, S. A., Barrionuevo, G., Benavides-Piccione, R., Burkhalter, A., Buzsáki, G., Cauli, B., DeFelipe, J., Fairén, A., Feldmeyer, D., Fishell, G., Fregnac, Y., Freund, T. F., Gardner, D., Gardner, E. P., Goldberg, J. H., Helmstaedter, M., Hestrin, S., … R. (2008). The Petilla Interneuron Nomenclature Group (PING): Petilla terminology: nomenclature of features of GABAergic interneurons of the cerebral cortex. *Nature Reviews Neuroscience,**9*(7), 557–568. 10.1038/nrn2402 10.1038/nrn2402 PMC2868386 18568015

[CR3] Ascoli, G. A., Krichmar, J. L., Scorcioni, R., Nasuto, S. J., Senft, S. L., & Krichmar, G. L. (2001). Computer generation and quantitative morphometric analysis of virtual neurons. *Anatomy and Embryology,**204*(4), 283–301. 10.1007/s004290100201 11720234 10.1007/s004290100201

[CR4] Belmonte, M. K., & Bourgeron, T. (2006). Fragile X syndrome and autism at the intersection of genetic and neural networks. *Nature Neuroscience,**9*(10), 1221–1225. 10.1038/nn1765 17001341 10.1038/nn1765

[CR5] Blackman, A. V., Grabuschnig, S., Legenstein, R., & Sjöström, P. J. (2014). A comparison of manual neuronal reconstruction from biocytin histology or 2-photon imaging: Morphometry and computer modeling. *Frontiers in Neuroanatomy, 8*. 10.3389/fnana.2014.00065 10.3389/fnana.2014.00065 PMC4092368 25071470

[CR6] Brodal, P. (2010). The Central Nervous System: Structure and Function, 4th ed. edn. *The central nervous system: Structure and function*. Oxford University Press, New York, NY, US. Pages: xiii, 591

[CR7] Budd, J. M. L., Kovács, K., Ferecskó, A. S., Buzás, P., Eysel, U. T., & Kisváday, Z. F. (2010). Neocortical Axon Arbors Trade-off Material and Conduction Delay Conservation. *PLOS Computational Biology,**6*(3), 1000711. 10.1371/journal.pcbi.1000711 10.1371/journal.pcbi.1000711 PMC2837396 20300651

[CR8] Cuntz, H., Forstner, F., Borst, A., & Häusser, M. (2010). One rule to grow them all: A general theory of neuronal branching and its practical application. *PLOS Computational Biology,**6*(8), 1000877. 10.1371/journal.pcbi.1000877 10.1371/journal.pcbi.1000877 PMC2916857 20700495

[CR9] Dickson, B. J. (2002). Molecular mechanisms of axon guidance. *Science,**298*(5600), 1959–1964. 10.1126/science.1072165 12471249 10.1126/science.1072165

[CR10] Economo, M. N., Winnubst, J., Bas, E., Ferreira, T. A., & Chandrashekar, J. (2019). Single-neuron axonal reconstruction: The search for a wiring diagram of the brain. *Journal of Comparative Neurology,**527*(13), 2190–2199. 10.1002/cne.24674 30859571 10.1002/cne.24674

[CR11] Gibson, D. A., & Ma, L. (2011). Developmental regulation of axon branching in the vertebrate nervous system. *Development.,**138*(2), 183–195. 10.1242/dev.046441 21177340 10.1242/dev.046441 PMC3005597

[CR12] Goodman, C. S., & Shatz, C. J. (1993). Developmental mechanisms that generate precise patterns of neuronal connectivity. *Cell,**72*, 77–98. 10.1016/S0092-8674(05)80030-3 8428376 10.1016/s0092-8674(05)80030-3

[CR13] Hegde, C., Indyk, P., & Schmidt, L. (2015). A nearly-linear time framework for graph-structured sparsity. In *Proceedings of the 32nd International conference on international conference on machine learning* - Volume 37. https://dl.acm.org/doi/10.5555/3045118.3045218

[CR14] Helmstaedter, M., Briggman, K. L., Turaga, S. C., Jain, V., Seung, H. S., & Denk, W. (2013). Connectomic reconstruction of the inner plexiform layer in the mouse retina. *Nature.,**500*(7461), 168–174. 10.1038/nature12346 23925239 10.1038/nature12346

[CR15] Hilgetag, C. C., & Zikopoulos, B. (2022). The highways and byways of the brain. *PLOS Biology,**20*(3), 3001612. 10.1371/journal.pbio.3001612 10.1371/journal.pbio.3001612 PMC9004754 35358176

[CR16] Iranmanesh, F., Narafshan, M. H., Golshan, M. (2021). A brain-based model of language instruction: from theory to practice. *Research and Development in Medical Education,**10*(1), 17–17. 10.34172/rdme.2021.017

[CR17] Ito, T., Hearne, L., Mill, R., Cocuzza, C., & Cole, M. W. (2020). Discovering the computational relevance of brain network organization. *Trends in Cognitive Sciences,**24*(1), 25–38. 10.1016/j.tics.2019.10.005 31727507 10.1016/j.tics.2019.10.005 PMC6943194

[CR18] Kanari, L., Dłotko, P., Scolamiero, M., Levi, R., Shillcock, J., Hess, K., Markram, H., & Arnaudon, A. (2024). A topological representation of branching neuronal morphologies. *Zenodo*. 10.5281/zenodo.10678146 10.1007/s12021-017-9341-1 PMC5797226 28975511

[CR19] Kanari, L., Arnaudon, A., Berchet, A., Coste, B., & Zisis, E. (2022a). NeuroTS. Blue Brain Project, EPFL. original-date: 2021-06-21T09:22:45Z. https://github.com/BlueBrain/NeuroTS. Accessed 19 Sept 2022

[CR20] Kanari, L., Dictus, H., Chalimourda, A., Arnaudon, A., Van Geit, W., Coste, B., Shillcock, J., Hess, K., & Markram, H. (2022). Computational synthesis of cortical dendritic morphologies. *Cell Reports,**39*(1), 110586. 10.1016/j.celrep.2022.110586 35385736 10.1016/j.celrep.2022.110586

[CR21] Kanari, L., Garin, A., & Hess, K. (2020). From trees to barcodes and back again: Theoretical and statistical perspectives. *Algorithms,**13*(12), 335. 10.3390/a13120335

[CR22] Karp, R. M. (1972). Reducibility among combinatorial problems. In: Miller, R.E., Thatcher, J.W., & Bohlinger, J.D. (Eds.) *Complexity of computer computations: Proceedings of a symposium on the complexity of computer computations* (pp. 85–103). Springer, Boston, MA. 10.1007/978-1-4684-2001-2_9

[CR23] Karube, F., Sári, K., & Kisvárday, Z. F. (2017). Axon topography of layer 6 spiny cells to orientation map in the primary visual cortex of the cat (area 18). *Brain Structure and Function,**222*(3), 1401–1426. 10.1007/s00429-016-1284-z 27539451 10.1007/s00429-016-1284-z PMC5368233

[CR24] Kasthuri, N., Hayworth, K. J., Berger, D. R., Schalek, R. L., Conchello, J. A., Knowles-Barley, S., Lee, D., Vázquez-Reina, A., Kaynig, V., Jones, T. R., Roberts, M., Morgan, J. L., Tapia, J. C., Seung, H. S., Roncal, W. G., Vogelstein, J. T., Burns, R., Sussman, D. L., Priebe, C. E., & Lichtman, J. W. (2015). Saturated reconstruction of a volume of neocortex. *Cell,**162*(3), 648–661. 10.1016/j.cell.2015.06.054 26232230 10.1016/j.cell.2015.06.054

[CR25] Kaufmann, W. E., & Moser, H. W. (2000). Dendritic anomalies in disorders associated with mental retardation. *Cerebral Cortex,**10*(10), 981–991. 10.1093/cercor/10.10.981 11007549 10.1093/cercor/10.10.981

[CR26] Kerstjens, S., Michel, G., & Douglas, R. J. (2022). Constructive connectomics: How neuronal axons get from here to there using gene-expression maps derived from their family trees. *PLOS Computational Biology,**18*(8), 1010382. 10.1371/journal.pcbi.1010382 10.1371/journal.pcbi.1010382 PMC9409546 36006873

[CR27] Koch, J. C., Bitow, F., Haack, J., d’Hedouville, Z., Zhang, J.-N., Tönges, L., Michel, U., Oliveira, L. M. A., Jovin, T. M., Liman, J., Tatenhorst, L., Bähr, M., & Lingor, P. (2015). Alpha-Synuclein affects neurite morphology, autophagy, vesicle transport and axonal degeneration in CNS neurons. *Cell Death & Disease,**6*(7), 1811–1811. 10.1038/cddis.2015.169 10.1038/cddis.2015.169 PMC4650722 26158517

[CR28] Koene, R. A., Tijms, B., Hees, P., Postma, F., Ridder, A., Ramakers, G. J. A., Pelt, J., & Ooyen, A. (2009). NETMORPH: A framework for the stochastic generation of large scale neuronal networks with realistic neuron morphologies. *Neuroinformatics,**7*(3), 195–210. 10.1007/s12021-009-9052-3 19672726 10.1007/s12021-009-9052-3

[CR29] Kollins, K. M., Bell, R. L., Butts, M., & Withers, G. S. (2009). Dendrites differ from axons in patterns of microtubule stability and polymerization during development. *Neural Development,**4*(1), 26. 10.1186/1749-8104-4-26 19602271 10.1186/1749-8104-4-26 PMC2717962

[CR30] Liu, Y., Bech, P., Tamura, K., Délez, L. T., Crochet, S., & Petersen, C. C. H. (2024) Cell class-specific long-range axonal projections of neurons in mouse whisker-related somatosensory cortices. *eLife, 13*. 10.7554/eLife.97602.1 10.7554/eLife.97602 PMC11469677 39392390

[CR31] Luczak, A. (2006). Spatial embedding of neuronal trees modeled by diffusive growth. *Journal of Neuroscience Methods,**157*(1), 132–141. 10.1016/j.jneumeth.2006.03.024 16690135 10.1016/j.jneumeth.2006.03.024

[CR32] Markram, H., Muller, E., Ramaswamy, S., Reimann, M. W., Abdellah, M., Sanchez, C. A., Ailamaki, A., Alonso-Nanclares, L., Antille, N., Arsever, S., Kahou, G. A. A., Berger, T. K., Bilgili, A., Buncic, N., Chalimourda, A., Chindemi, G., Courcol, J.-D., Delalondre, F., Delattre, V., … Schürmann, F. (2015). Reconstruction and simulation of neocortical microcircuitry. *Cell.,**163*(2), 456–492. 10.1016/j.cell.2015.09.029 10.1016/j.cell.2015.09.029 26451489

[CR33] Mateus, J. C., Sousa, M. M., Burrone, J., & Aguiar, P. (2024) Beyond a transmission cable—new technologies to reveal the richness in axonal electrophysiology. *Journal of Neuroscience, 44*(11). 10.1523/JNEUROSCI.1446-23.2023 10.1523/JNEUROSCI.1446-23.2023 PMC10941245 38479812

[CR34] Moolman, D. L., Vitolo, O. V., Vonsattel, J.-P.G., & Shelanski, M. L. (2004). Dendrite and dendritic spine alterations in Alzheimer models. *Journal of Neurocytology,**33*(3), 377–387. 10.1023/B:NEUR.0000044197.83514.64 15475691 10.1023/B:NEUR.0000044197.83514.64

[CR35] Ofer, N., Shefi, O., & Yaari, G. (2017). Branching morphology determines signal propagation dynamics in neurons. *Scientific Reports,**7*(1), 8877. 10.1038/s41598-017-09184-3 28827727 10.1038/s41598-017-09184-3 PMC5567046

[CR36] Osten, P., & Margrie, T. W. (2013). Mapping brain circuitry with a light microscope. *Nature Methods,**10*(6), 515–523. 10.1038/nmeth.2477 23722211 10.1038/nmeth.2477 PMC3982327

[CR37] Petkantchin, R., Berchet, A., Peng, H., Markram, H., & Kanari, L. (2024) Generating brain-wide connectome using synthetic axonal morphologies. *bioRxiv*. 10.1101/2024.10.04.616605 10.1038/s41467-025-62030-3 PMC12271523 40676034

[CR38] Rama, S., Zbili, M., & Debanne, D. (2018). Signal propagation along the axon. *Current Opinion in Neurobiology,**51*, 37–44. 10.1016/j.conb.2018.02.017 29525575 10.1016/j.conb.2018.02.017

[CR39] Ramón y Cajal, S. (1911) Histologie du Système Nerveux de L’homme & des Vertébrés, Ed. française rev. & mise à jour par l’auteur, tr. de l’espagnol par l. azoulay. edn. Maloine, Paris. 10.5962/bhl.title.48637 . Pages: 1-1012

[CR40] Reimann, M. W., Bolaños-Puchet, S., Courcol, J.-D., Santander, D. E., Arnaudon, A., Coste, B., Delalondre, F., Delemontex, T., Devresse, A., Dictus, H., Dietz, A., Ecker, A., Favreau, C., Ficarelli, G., Gevaert, M., Herttuainen, J., Isbister, J. B., Kanari, L., Keller, D., ... Ramaswamy, S. (2024). Modeling and Simulation of Neocortical Micro- and Mesocircuitry. *Part I: Anatomy. eLife,**13*. 10.7554/eLife.99688.2 10.7554/eLife.99688 PMC12818870 41556767

[CR41] Sakai, N., & Kaprielian, Z. (2012) Guidance of longitudinally projecting axons in the developing central nervous system. *Frontiers in Molecular Neuroscience, 5*. 10.3389/fnmol.2012.00059. 10.3389/fnmol.2012.00059 PMC3343325 22586366

[CR42] Shin, J. H., Song, M., Paik, S.-B., & Jung, M. W. (2020). Spatial organization of functional clusters representing reward and movement information in the striatal direct and indirect pathways. *Proceedings of the National Academy of Sciences,**117*(43), 27004–27015. 10.1073/pnas.2010361117 10.1073/pnas.2010361117 PMC7604453 33055217

[CR43] Srivastava, D. P., Woolfrey, K. M., Jones, K. A., Anderson, C. T., Smith, K. R., Russell, T. A., Lee, H., Yasvoina, M. V., Wokosin, D. L., Ozdinler, P. H., Shepherd, G. M. G., & Penzes, P. (2012). An autism-associated variant of epac2 reveals a role for ras/epac2 signaling in controlling basal dendrite maintenance in mice. *PLOS Biology,**10*(6), 1001350. 10.1371/journal.pbio.1001350 10.1371/journal.pbio.1001350 PMC3383751 22745599

[CR44] Thiebaut de Schotten, M., ffytche, D.H., Bizzi, A., Dell’Acqua, F., Allin, M., Walshe, M., Murray, R., Williams, S. C., Murphy, D. G. M., & Catani, M. (2011). Atlasing location, asymmetry and inter-subject variability of white matter tracts in the human brain with MR diffusion tractography. *NeuroImage,**54*(1), 49–59. 10.1016/j.neuroimage.2010.07.055 10.1016/j.neuroimage.2010.07.055 20682348

[CR45] Thomson, A. M., & Armstrong, W. E. (2011). Biocytin-labelling and its impact on late 20th century studies of cortical circuitry. *Brain Research Reviews,**66*(1), 43–53. 10.1016/j.brainresrev.2010.04.004 20399808 10.1016/j.brainresrev.2010.04.004 PMC2949688

[CR46] Torben-Nielsen, B., & Cuntz, H. (2014). Introduction to Dendritic Morphology. In Cuntz, H., Remme, M.W.H., Torben-Nielsen, B. (eds.) *The computing dendrite: From structure to function* (pp. 3–22). Springer, New York, NY. 10.1007/978-1-4614-8094-5_1

[CR47] Venkadesh, S., Santarelli, A., Boesen, T., Dong, H.-W., & Ascoli, G. A. (2023). Combinatorial quantification of distinct neural projections from retrograde tracing. *Nature Communications,**14*(1), 7271. 10.1038/s41467-023-43124-2 37949860 10.1038/s41467-023-43124-2 PMC10638408

[CR48] Wang, Q., Ding, S.-L., Li, Y., Royall, J., Feng, D., Lesnar, P., Graddis, N., Naeemi, M., Facer, B., Ho, A., Dolbeare, T., Blanchard, B., Dee, N., Wakeman, W., Hirokawa, K. E., Szafer, A., Sunkin, S. M., Oh, S. W., Bernard, A., … L. (2020). The Allen Mouse Brain Common Coordinate Framework: A 3D Reference Atlas. *Cell,**181*(4), 936–95320. 10.1016/j.cell.2020.04.007 10.1016/j.cell.2020.04.007 PMC8152789 32386544

[CR49] Wang, N., Anderson, R. J., Badea, A., Cofer, G., Dibb, R., Qi, Y., & Johnson, G. A. (2018). Whole mouse brain structural connectomics using magnetic resonance histology. *Brain Structure and Function,**223*(9), 4323–4335. 10.1007/s00429-018-1750-x 10.1007/s00429-018-1750-x PMC7053251 30225830

[CR50] Wheeler, D. W., Banduri, S., Sankararaman, S., Vinay, S., & Ascoli, G. A. (2024). Unsupervised classification of brain-wide axons reveals the presubiculum neuronal projection blueprint. *Nature Communications,**15*(1), 1555. 10.1038/s41467-024-45741-x 38378961 10.1038/s41467-024-45741-x PMC10879163

[CR51] Winnubst, J., Bas, E., Ferreira, T. A., Wu, Z., Economo, M. N., Edson, P., Arthur, B. J., Bruns, C., Rokicki, K., Schauder, D., Olbris, D. J., Murphy, S. D., Ackerman, D. G., Arshadi, C., Baldwin, P., Blake, R., Elsayed, A., Hasan, M., Ramirez, D., . . . Chandrashekar, J. (2019). Reconstruction of 1,000 Projection Neurons Reveals New Cell Types and Organization of Long-Range Connectivity in the Mouse Brain. *Cell,**179*(1), 268–28113. 10.1016/j.cell.2019.07.042 10.1016/j.cell.2019.07.042 PMC6754285 31495573

[CR52] Winter, C. C., Jacobi, A., Su, J., Chung, L., Velthoven, C. T. J., Yao, Z., Lee, C., Zhang, Z., Yu, S., Gao, K., Duque Salazar, G., Kegeles, E., Zhang, Y., Tomihiro, M. C., Zhang, Y., Yang, Z., Zhu, J., Tang, J., Song, X., ... He, Z. (2023). A transcriptomic taxonomy of mouse brain-wide spinal projecting neurons. *Nature*, *624*(7991), 403–414. 10.1038/s41586-023-06817-8 10.1038/s41586-023-06817-8 PMC10719099 38092914

[CR53] Zhou, J., Zhang, Z., Wu, M., Liu, H., Pang, Y., Bartlett, A., Peng, Z., Ding, W., Rivkin, A., Lagos, W. N., Williams, E., Lee, C.-T., Miyazaki, P. A., Aldridge, A., Zeng, Q., Salinda, J. L. A., Claffey, N., Liem, M., Fitzpatrick, C., ... Callaway, E. M. (2023). Brain-wide correspondence of neuronal epigenomics and distant projections. *Nature*, *624*(7991), 355–365. 10.1038/s41586-023-06823-w 10.1038/s41586-023-06823-w PMC10719087 38092919

[CR54] Zingg, B., Hintiryan, H., Gou, L., Song, M. Y., Bay, M., Bienkowski, M. S., Foster, N. N., Yamashita, S., Bowman, I., Toga, A. W., & Dong, H.-W. (2014). Neural Networks of the Mouse Neocortex. *Cell,**156*(5), 1096–1111. 10.1016/j.cell.2014.02.023 10.1016/j.cell.2014.02.023 PMC4169118 24581503

[CR55] Zubler, F., & Douglas, R. (2009). A framework for modeling the growth and development of neurons and networks. *Frontiers in Computational Neuroscience,**3*. 10.3389/neuro.10.025.2009 10.3389/neuro.10.025.2009 PMC2784082 19949465

